# Considering cell volume in dopant screening for improving Li-ion mobility in an amorphous LiPON solid-state electrolyte: an *ab initio* study†

**DOI:** 10.1039/d3ra00557g

**Published:** 2023-05-10

**Authors:** Heechae Choi, Seulgi Ji, Haneol Cho, Chansoo Kim, Patrick Joohyun Kim, Hyunjung Park, Junghyun Choi

**Affiliations:** a Department of Chemistry, Xi'an Jiaotong-Liverpool University Suzhou Industrial Park 215123 Suzhou China; b Theoretical Materials & Chemistry Group, Institute of Inorganic Chemistry, University of Cologne Greinstr. 6 50939 Cologne Germany; c Korea Institute of Science and Technology Hwarangro 14 Gil 5 136-791 Seoul Korea; d Department of Applied Chemistry, Kyungpook National University Daegu 41566 Korea; e Department of Materials Science and Engineering, Chosun University Gwangju 61452 Korea; f Energy Storage Materials Center, Korea Institute of Ceramic Engineering and Technology Jinju 52851 Korea jchoi@kicet.re.kr

## Abstract

Engineering of solid electrolytes of Li-ion batteries is carried out for achieving high levels of ionic conductivity and preserving low levels of electrical conductivity. Doping metallic elements into solid electrolyte materials composed of Li, P, and O is quite challenging due to instances of possible decomposition and secondary phase formation. To accelerate the development of high-performance solid electrolytes, predictions of thermodynamic phase stabilities and conductivities are necessary, as they would avoid the need to carry out exhaustive trial-and-error experiments. In this study, we demonstrated theoretical approach to increase the ionic conductivity of amorphous solid electrolyte by doping: cell volume-ionic conductivity relation. Using density functional theory (DFT) calculations, we examined the validity of the hypothetical principle in predicting improvements in stability and ionic conductivity with 6 candidate doping elements (Si, Ti, Sn, Zr, Ce, Ge) in a quaternary Li–P–O–N solid electrolyte system (LiPON) both in crystalline and amorphous phases. The doping of Si into LiPON (Si–LiPON) was indicated to stabilize the system and enhance ionic conductivity based on our calculated doping formation energy and cell volume change. The proposed doping strategies provide crucial guidelines for the development of solid-state electrolytes with enhanced electrochemical performances.

## Abstract 2

Engineering of solid electrolytes of Li-ion batteries is carried out for achieving high levels of ionic conductivity and preserving low levels of electrical conductivity.

## Graphical abstract

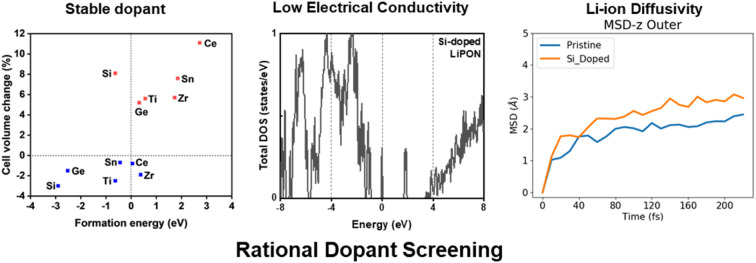

## Introduction

In recent years, electrochemical energy storage devices have gained much attention due to the high demand for eco-friendly electric vehicles (EVs) or portable electronic devices, which help to mitigate climate change by using zero-emission energy sources.^1–4^ The lithium-ion battery (LIB) features energy and power density and cycling durability higher than those of any other energy-storage devices. The current generation of LIB systems mainly uses liquid electrolytes. However, large-scale applications of LIBs such as in EVs are hindered by safety issues such as the electrolyte flammability.^5,6^ In addition, the inevitable formation of Li dendrites causes the efficiency of LIBs to decrease to unsatisfactory levels, leading to chemical short circuits.^7^

All-solid-state batteries (ASSBs) are considered the next-generation battery system, in which the flammable liquid electrolytes of the conventional LIB systems are substituted with non-flammable solid electrolytes (SEs).^8^ To produce ideal LIBs, the SEs of ASSBs must meet many requirements, such as high energy density, extended cycle life, high safety, a wide operating temperature range, and low electrical conductivity.^9–11^ High ionic conductivity of electrolytes significantly contributes to the performance of LIBs, as the speed of charging/discharging through movement or intercalations of Li ions in the electrolyte and electrodes mainly determines the market value of the LIBs. However, it is still challenging to derive a rational design for increasing the ionic conductivity of SEs to the needs of the market.^12^ Therefore, carrying out systematic studies to design and develop new SE materials having enhanced ionic conductivity and low electrical conductivity is in high demand.

Lithium phosphorus oxynitride (LiPON) is one of the most commonly used SEs for LIBs since its initial discovery in the 1990s at the Oak Ridge National Laboratory.^13^ LiPON shows several advantages such as less sensitivity to air than displayed by other SEs, for example, Li_7_La_3_Zr_2_O_12_ (LLZO), a negligible electronic conductivity, a wide electrochemical window, and high stability in the presence of lithium metal.^14^ The high stability of LiPON in the presence of Li metal anode is due to the formation of a solid electrolyte interphase (SEI) film. Despite the outstanding properties of LiPON, the ionic conductivity of available forms of LiPON is poor (10^−6^ to 10^−8^ S cm^−1^) and improving this conductivity remains challenging, hence impeding the widespread use of LiPON as an SE for LIBs.^15,16^ Doping foreign element atoms into LiPON, however, could provide multiple advantages, including improving its electrochemical performance and processability. However, there are only a few reports on doping strategies for LiPON.^17,18^ In order to have LiPON achieve electrochemical performance measures comparable to those of other SEs, an in-depth investigation of appropriate dopants for LiPON needs to be conducted.

In this work, we aimed to rationally investigate LiPON SE dopants that can best enhance the ionic conductivity of this SE while preserving a high electrical resistance, and carried out this investigation by performing density functional theory (DFT) calculations. We hypothesized that using a tetravalent metal (TM) for doping, which has been demonstrated with only a couple of elements,^19,20^ may enhance the ionic conductivity of LiPON since TM substitution has been indicated to break the tetrahedral oxygen bonds in PO_4_, and widen the Li migration channel. We first modeled the amorphized pristine LiPON and LiPONs doped with tetravalent cations (TM–LiPONs) to predict the metallicity, cell volumes, and thermal stability levels of the TM–LiPONs compared to those of the undoped LiPON system. In addition, we further demonstrated the validity of the cell-volume-based screening scheme with *ab initio* molecular dynamics (AIMD) simulations, which directly showed the Li mobility within undoped and doped LiPON having different cell volumes.

## Computational method

DFT calculations and AIMD simulations were carried out using the projected augmented wave (PAW) method with the Vienna *Ab initio* Simulation Package (VASP) to study the interaction between valence and core electrons. All DFT calculations were conducted with the general gradient approximation (GGA) and the Perdew–Burke–Ernzerhof (PBE) exchange–correlation functional.^21,22^ Optimization of cell parameters was achieved using gamma-centered k-points in a 2 × 2 × 1 grid for a cell for Brillouin zone sampling, and a kinetic energy cut-off of 400 eV was used. The amorphized LiPON and TM(iv)–LiPON supercells were prepared using *ab initio* molecular dynamics (AIMD) simulations and relaxations.^23^ The temperature for the AIMD was set to 3000 K and the AIMD simulations were carried out for 5 ns. A plane-wave energy cutoff of 300 eV and a *Γ*-centered 1 × 1 × 1 *k*-point grid was used. We also calculated a root-mean-squared displacement (RMSD) to evaluate Li-ion mobilities for 240 fs at 1000 K using eqn (1), in which *N*, *x*_*t*_ − *x*_0_, and *t* represent the total number of Li ions in the system, the displacement of Li ions in Å, and time.1
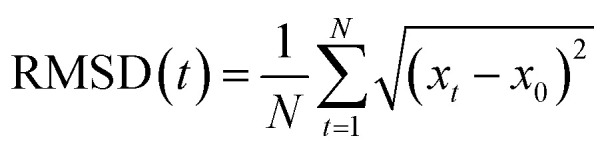


**Fig. 1:**
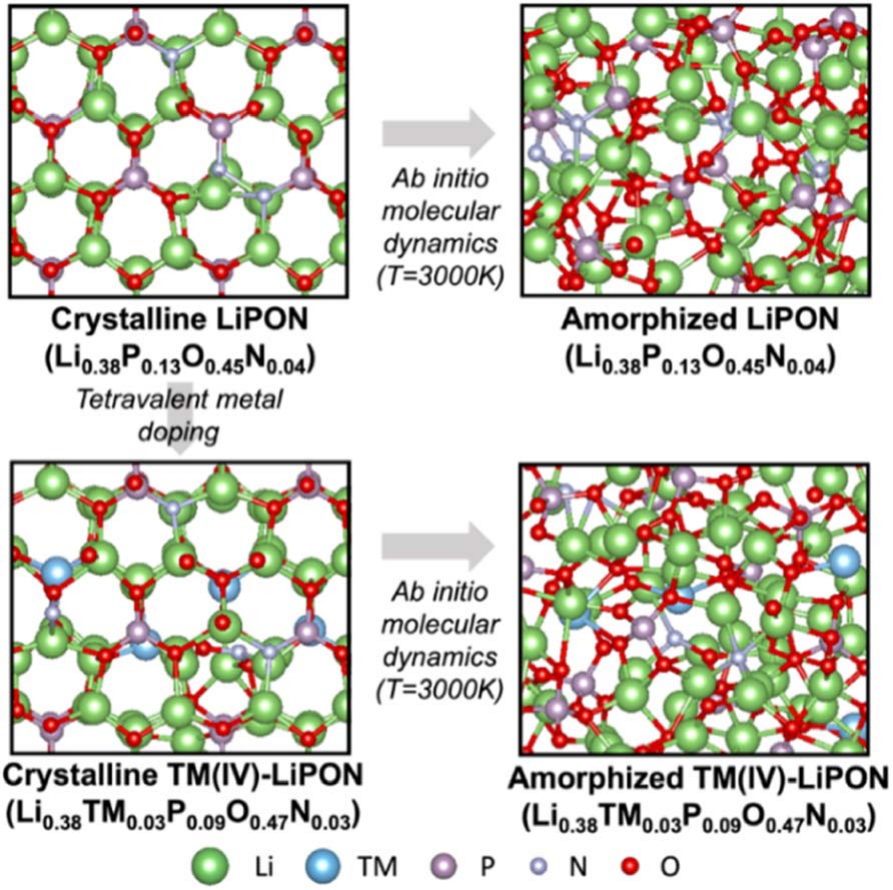
Depictions of the atomic structures of crystalline and amorphous LiPON and TM-doped LiPON.

The supercell of LiPON (Li_0.38_P_0.17_O_0.45_N_0.04_) consisted of 47 Li, 16 P, 56 O, and 5 N atoms, and that of TM(iv)–LiPONs (Li_0.38_TM_0.03_P_0.13_O_0.45_N_0.03_) consisted of 48 Li, 4 TM(iv), 12 P, 60 O, and 4 N atoms (Fig. 1). The radial distribution functions (*g*(*r*)) were plotted to show well-amorphized LiPON and TM(iv)–LiPONs after thermal heating using AIMD simulations, as shown in Fig. S1.†

## Results and discussion

**Fig. 2:**
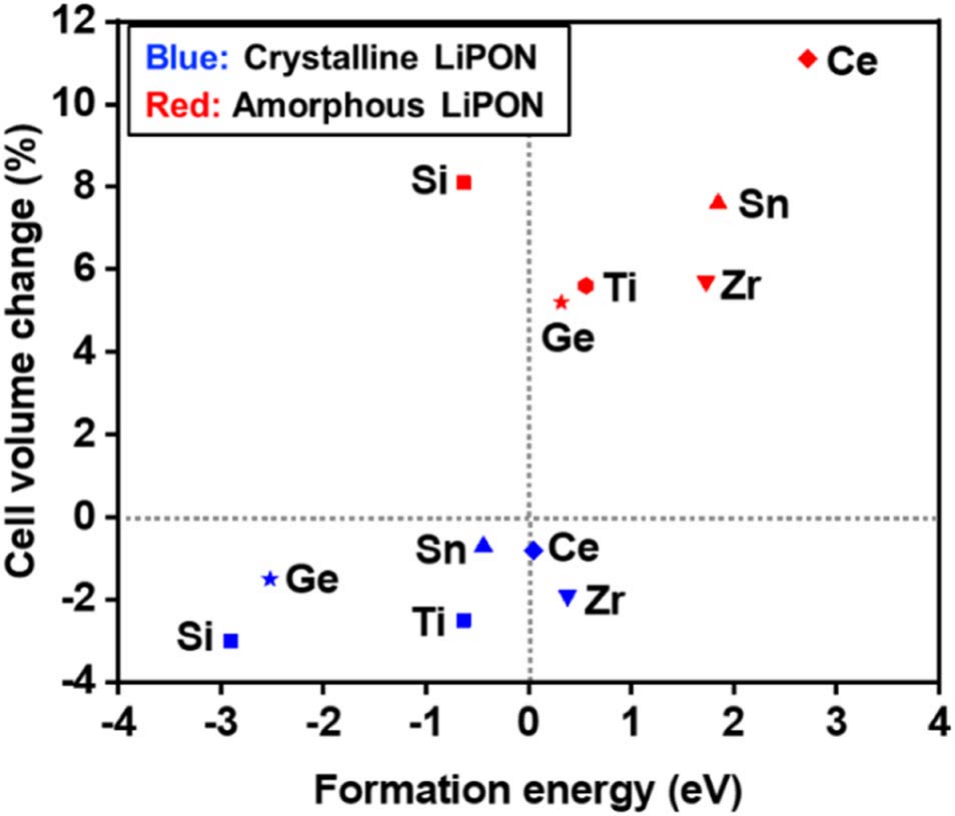
Calculated formation energies and cell volume changes of dopants in LiPON. The blue and red marks indicate the data for the crystalline and amorphous LiPON host matrices, respectively.

The calculated heats of formation of crystalline and amorphous LiPON were −1.06 eV per atom and −0.45 eV per atom, respectively. Therefore, our crystalline and amorphous LiPON supercell models satisfied the thermal stability conditions, consistent with experiments.^22,23^ We predicted the thermal stability levels and the cell volumes of doped LiPON under the hypothetical assumption that the change of cell volume of LiPON would be related to ionic conductivity. In recent studies combining DFT and experiment, the increase in unit cell volumes of crystalline oxide or phosphide systems were indicated to be beneficial to rapid migration of Li ions.^24,25^ In Fig. 2, the doping formation energy and the cell volume change for the theoretical crystalline and amorphous LiPON structures are presented. Due to LiPON becoming amorphized after a few cycles of operations, it was necessary to consider the thermodynamic stability of the amorphous phase. Doping Si, Ge, Ti, and Sn into a crystal of LiPON and having them substitute for phosphorous were indicated to be energetically favorable, as indicated by the negative values of formation energy (Fig. 2). However, all the doping elements considered in this study were indicated to reduce the LiPON crystal unit cell volume—but, interestingly, to increase the amorphous LiPON cell volume, indicating the ability of the dopants to enhance the Li ion conductivity.

Lee *et al.* reported that, in their experiments, doping Ti into amorphous LiPON enhanced Li^+^ ionic conductivity,^17^ quite consistent with our theoretical calculations on the effect of Ti-doping on the ionic conductivity. However, due to the poor energetic stability of Ti-doped LiPON, compared to that of undoped LiPON, more rapid formation of secondary phases and worse cyclability (shorter lifetime) were expected. Our computations showed only doping of Si into amorphous LiPON to be energetically favorable. Therefore, Si-doped LiPON can have stability and ionic conductivity levels superior to those of undoped amorphous LiPON if the electrical resistance is kept large enough.

**Fig. 3:**
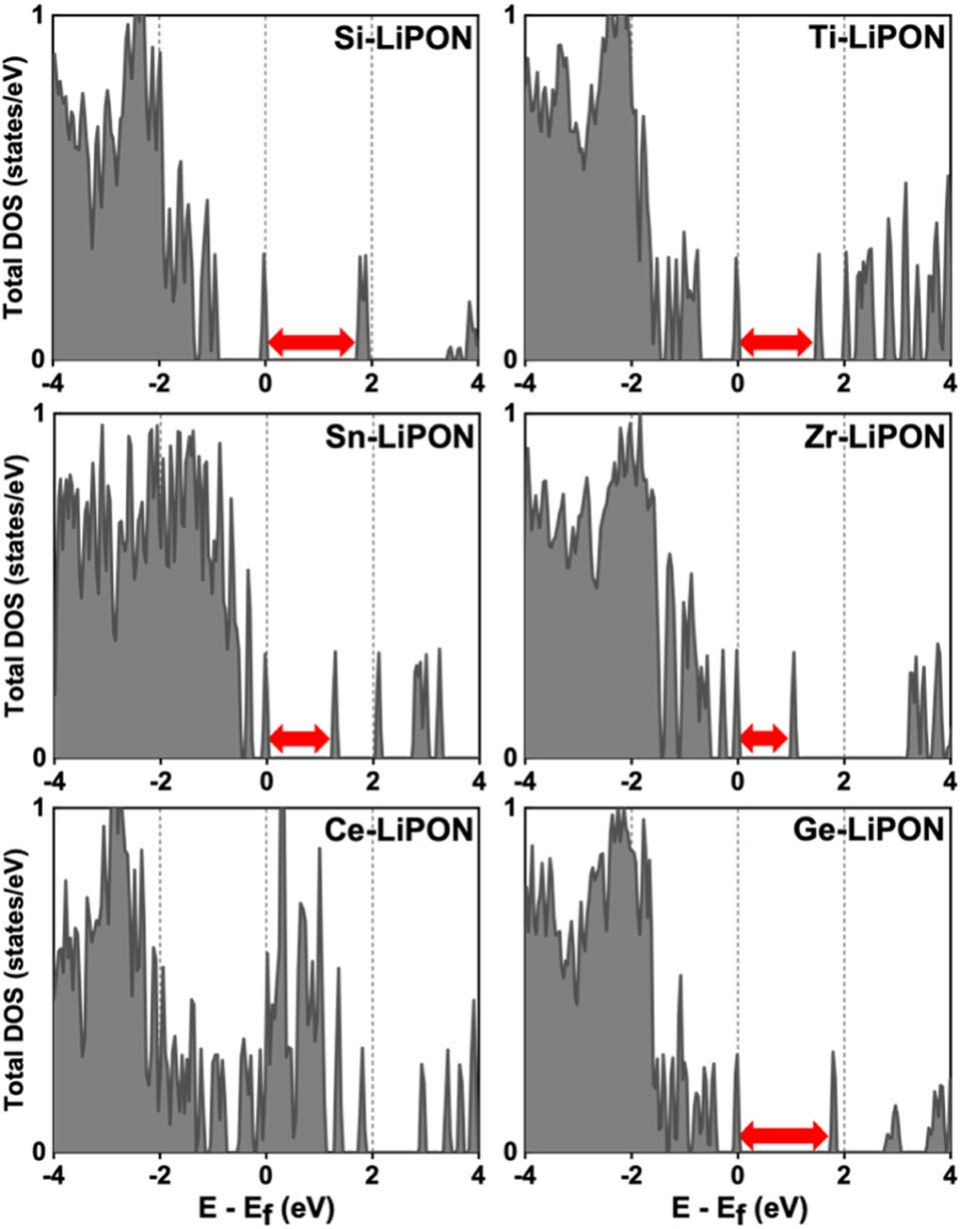
Calculated electron density of states (DOS) of TM-doped amorphous-phase LiPON.

In many cases, engineering materials by doping or amorphizing them greatly increases their electrical conductivity by forming many defect sites near their conduction bands.^26,27^ Therefore, considerations of the metallicity of new synthesized solid electrolyte materials are essential in theoretical designs of these materials.^28^ Calculating electron density of states (DOS) when carrying out DFT calculations is a direct and simple way to predict the electrical conductivity of a candidate material. Fig. 3 shows the calculated electron DOS of TM-doped LiPON. This calculation was carried out to investigate the possibility of making amorphous LiPON more metallic by doping metals into it. It is important that the DOS of a candidate material not have populations of electrons at or near the Fermi energy (marked as 0 eV in Fig. 3) in order for the material to be used as a solid-state electrolyte. The Si-, Ti-, Sn-, Zr, and Ge-doped LiPON materials were calculated to have energy gaps between the highest occupied and lowest unoccupied electron states of 1.9, 1.7, 1.5, 1.2, and 1.8 eV, respectively. Considering the general tendency of a larger gap to induce a larger electrical resistivity, these calculations suggested that Si-doped LiPON would display the highest electrical resistivity. Of the six dopants into LiPON considered in this study, Ce was the only one that when doped into amorphous-phase LiPON would, according to our calculations, make the system completely metallic. Despite Ce doping being calculated to increase the volume of LiPON (Fig. 2) to the greatest extent (by 11.1%), it would be useless for amorphous LiPON solid electrolyte due to the metallicity issue. Si-doping, which was found to enhance the thermal stability and increase the cell volume of amorphous LiPON, also induces formation of deep defect levels. The occupied defect level close to the valence band maximum (VBM) of amorphous LiPON was calculated to be very much localized, and hence not related to electrical conductivity. Ti-doping was calculated to slightly narrow the band gap of LiPON, consistent with the experimental results of reduced impedance.^17^

**Fig. 4:**
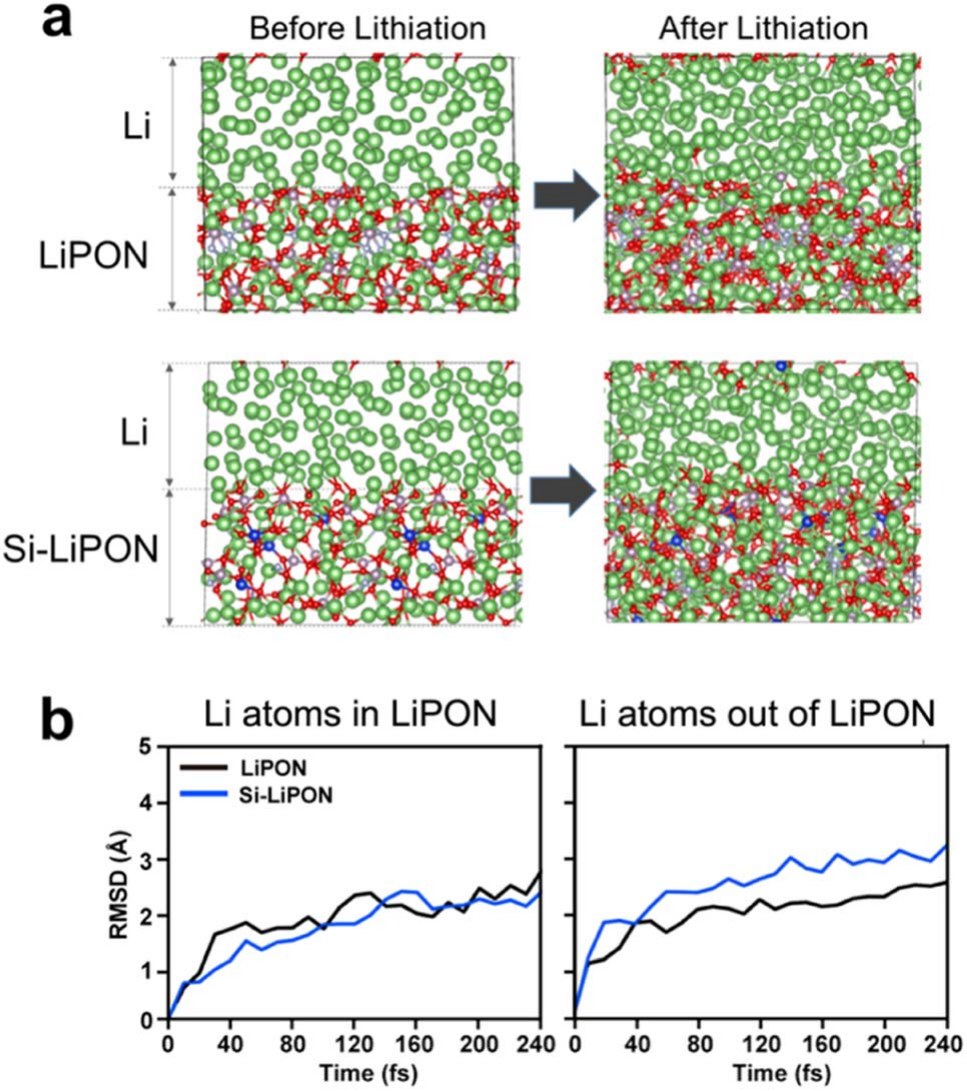
(a) Atomic structures of the Li/LiPON and Li/Si–LiPON interfaces before and after they were subjected to AIMD simulations at a temperature of 1000 K for 10 fs time laps and (b) the calculated RSMD values for Li atoms within and outside of LiPON and Si–LiPON solids. The blue and orange lines show the results for LiPON and Si–LiPON, respectively.

**Fig. 5:**
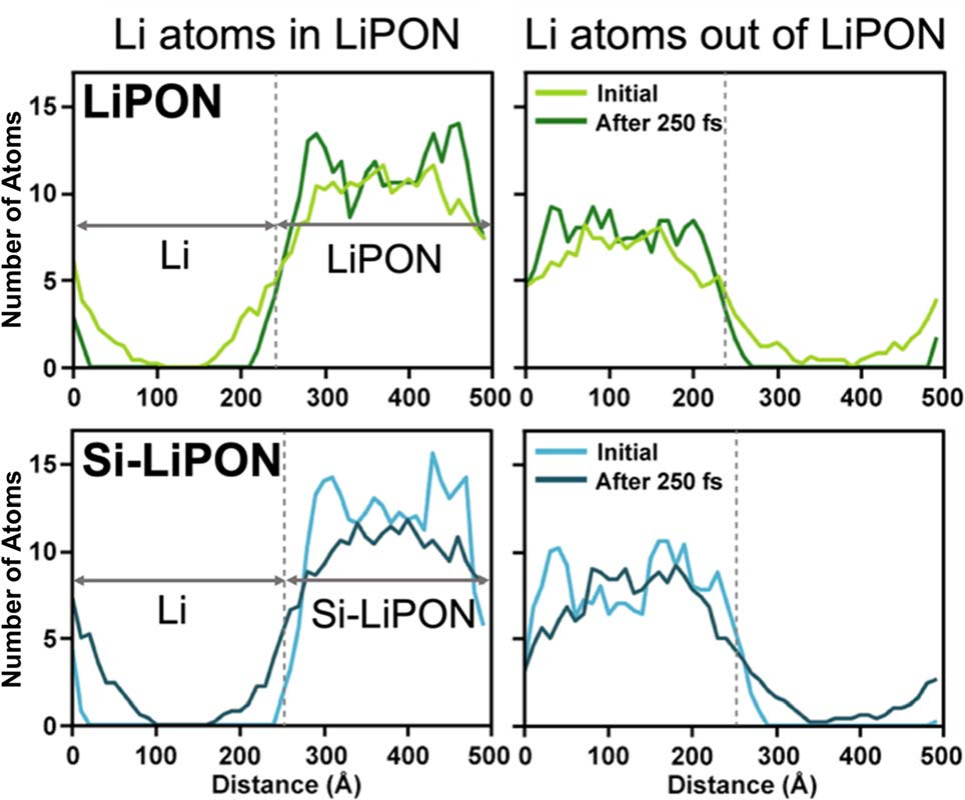
Plots showing the number of Li atoms inside and outside of LiPON and Si–LiPON junctions before and after (250 fs) lithiation at a temperature of 1000 K.

In Fig. 4, we present the results of AIMD simulations performed to demonstrate the validity of cell-volume-based dopant screening. Using AIMD simulation at a temperature of 1000 K, we calculated the Li mobility in undoped and Si-doped LiPON models (Fig. 4a). The calculated RSMD values for Li atoms showed a much faster Li-ion migration for Si–LiPON than for the undoped material (Fig. 4b). We analysed the RSMD values of Li atoms within the amorphous LiPON structure and of Li atoms outside of but coming into LiPON. Interestingly, the RSMD values for Li atoms forming the amorphous LiPON structure were not significantly altered with doping of Si into the LiPON matrix. However, in the simulations, Li atoms penetrated much faster into the Si–LiPON structure than into the LiPON structure. The numbers of Li atoms within and outside of LiPON and Si–LiPON were similar (2.17% difference). Therefore, the enhanced mobility of Li in LiPON resulting from Si doping was purely from the lattices of LiPON and Si–LiPON (Fig. 5).

## Conclusions

In the current work, based on DFT calculations and AIMD simulations, we suggested a rational strategy for examining the validity of a design principle for amorphous SE: enhancing Li ion mobility by carrying out doping to increase cell volume. First, considering the thermal stability and cell volume change of amorphous LiPON, we suggested doping of Si into LiPON, which to the best of our knowledge has never been suggested before for LiPON-based SE material systems. With calculations of energy, cell volume, and electron DOS, and analyses of our AIMD simulations, we demonstrated that the cell volume expansion can be used as an indicator for SE material design. In this regard, we suggest that doping Si into amorphous LiPON SE is beneficial due to the theoretically predicted increase in Li-ion mobility and good level of electrical resistivity. Our theoretical predictions on doping of Ti into amorphous LiPON were in good agreement with previous experimental work. We expect this theoretical work to inspire investigators working on computation-driven SE screening to utilize cell volume as a key indicator for dopant and composition screening.

## Conflicts of interest

There are no conflicts to declare.

## Supplementary Material

RA-013-D3RA00557G-s001
