# Supplementary material for: Considering cell volume in dopant screening for improving Li-ion mobility in an amorphous LiPON solid-state electrolyte: an ab initio study
Source: RSC Adv. 2023 May 10;13(21):14379–83. doi: 10.1039/d3ra00557g (PMC10171259; doi:10.1039/d3ra00557g)
Supplement: RA-013-D3RA00557G-s001 [file RA-013-D3RA00557G-s001.pdf]

Electronic Supplementary Material (ESI) for RSC Advances.
This journal is © The Royal Society of Chemistry 2023

Supporting Information

Cell Volume-Wise Dopant Screening for Amorphous LiPON Solid State Electrolyte toward Improved Li-ion Mobility: An ab initio study

Heechae Choi,[a,b] Seulgi Ji,[b] Haneol Cho,[c] Chansoo Kim,[c] Patrick Joohyun Kim,[d] Hyunjung Park[e] and Junghyun Choi,[f,*]

[a]*Department of Chemistry, Xi'an Jiaotong–Liverpool University, Suzhou Industrial Park, 215123, Suzhou, China*
[b]*Theoretical Materials & Chemistry Group, Institute of Inorganic Chemistry, University of Cologne, Greinstr. 6, 50939, Cologne, Germany*
[c]*Korea Institute of Science and Technology, Hwarangro 14Gil 5, 136-791, Seoul, Korea*
[d]*Department of Applied Chemistry, Kyungpook National University, Daegu, 41566, Korea*
[e]*Department of Materials Science and Engineering, Chosun University, Gwangju, 61452, Korea*
[f]*Energy Storage Materials Center, Korea Institute of Ceramic Engineering and Technology, Jinju, 52851, Korea. E-mail: jchoi@kicet.re.kr*

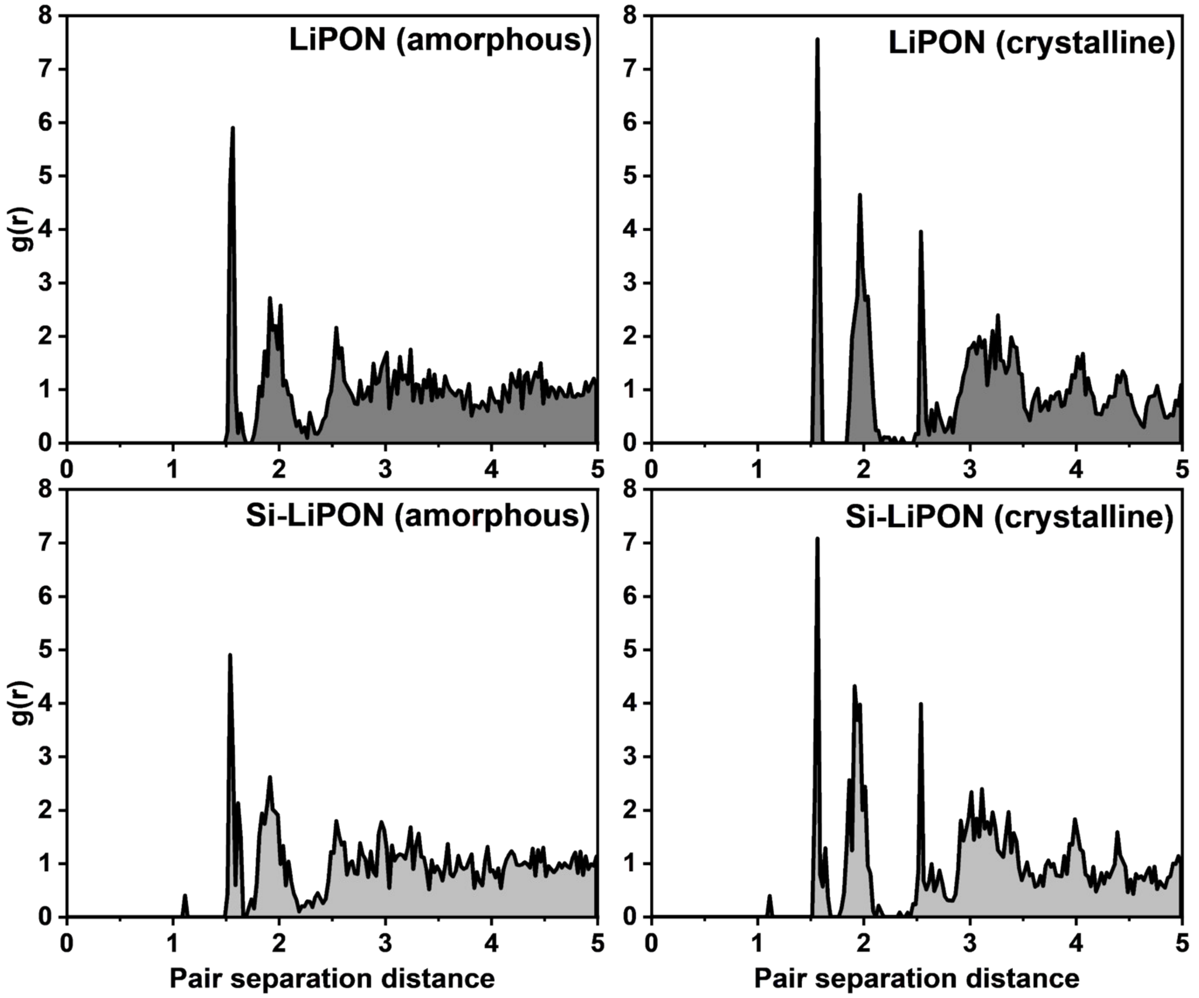

Figure S1. The radial distribution function g(r) of amorphous LiPON, Si-LiPON and crystalline LiPON, Si-LiPON.

The radial distribution function g(r) represents the mean number of atoms in a shell of width dr at distance r can be obtained by using following equation,

$$g(r) = p(r)(\rho 4\pi r^2\, dr)$$

where p(r) is the pair correlation function and ρ represent the atom density.[1] Crystalline LiPON and Si-LiPON showed more sharp lines than amorphous LiPON and Si-LiPON, indicating amorphization was well performed after ab initio molecular dynamics (AIMD) simulations at 3000 K.

Reference

(1) Lee, I.-H.; Chang, K. J. Crystal Structure Prediction in a Continuous Representative Space. *Computational Materials Science* **2021**, *194*, 110436. https://doi.org/10.1016/j.commatsci.2021.110436.
